# Progress in Surgery

**Published:** 1898-12-03

**Authors:** 


### PROGRESS IN SURGERY.

### SURGICAL TREATMENT OF EPILEPSY.

In reporting a series of 14 cases, illustrative of the surgical treatment of epilepsy, Andrew J. McCosh, of New York,[1] takes the opportunity of summarising the current views on this particular aspect of cerebral surgery. While admitting that the present feeling of uncertainty, even of scepticism, regarding the value of surgical interference in intracranial conditions is to a certain extent justified by the experience of the earlier operations in this line, the author thinks that the pendulum of surgical opinion has perhaps swung too far in the direction of pessimism.

One great desideratum to the formation of a correct estimate of the value of such procedures is wanting, namely, reliable statistics—that is statistics of *all* the operations done, more especially by surgeons of wide experience and outstanding skill in cranial surgery. Too often isolated successful cases are reported, the negative or disastrous results remaining in oblivion. Reports of the ultimate result of those cases which do recover from the operation should now be forthcoming. Referring to cerebral tumours, the author says he believes that, could all operations be tabulated, the death-rate would be at least 75 per cent.

In the case of epilepsy the operation mortality is certainly much lower, probably 5 or 6 per cent., but the ultimate and permanent cure of the patient is still a rare result. Bergmann is only able to report two cures out of 50 cases operated upon; Mason collected at random 70 cases, of which only 4·03 per cent. were still well at the end of three years; Sachs and Gerster had three cures out of 19 patients; three years being taken as the limit. Results published within a period of three years are certainly unreliable, as recurrence often takes place up to that time. At the same time, the condition for which the operation is undertaken is so desperate, and the outlook so black, that a certain proportion of selected cases should get the chance which surgery can offer.

Failure in the majority of cases is not due to defective technique, but to other difficulties which are less readily surmounted, such as the difficulty of accurately localising lesions situated elsewhere than in the motor areas; the inaccessibility of certain parts of the brain; the character of certain growths and lesions being such as to preclude the possibility of their removal; the leaving of post-operation lesions which perpetuate the symptoms; and the fact that secondary and irremovable changes are often produced by the primary lesion before operation is undertaken.

**Indications for Operation.**—The cases subjected to operation must be carefully selected. The author says that in general operation is indicated: (1) In all cases of focal or partial epilepsy (Jacksonian), where the convulsive movements or paralyses are limited to a particular group of muscles; (2) in all cases where the epilepsy, be it general or partial, has followed, and is apparently caused by a depression in the skull, the result of a traumatism; the more recent the injury is the better is the prospect of recovery, because of the diminished time for the development of secondary lesions; (3) in many cases where a severe head injury —even though there be no external evidence—has been followed by a partial epilepsy, and where the "signal symptom" indicates a definite area in the brain. It is often a matter for debate in deciding where to open the skull, whether the position of the injury to the skull or the brain lesion, as indicated by the "signal symptom," should be the guide. Opinions differ. The surgeon prefers as a rule the surgical indication, the neurologist the brain symptom—and each is frequently wrong.

**Methods of Operation.**—The author refers to the oft-debated subject of how best to open the skull, once an operation has been decided upon. The relative merits and drawbacks of the trephine, bone forceps, chisel and mallet, saw and burr are once more paraded, and the outcome is that there is little to choose between them. Success depends more upon accuracy of diagnosis than upon technique.

**Results in Author's Series of Cases.**—Since 1890 the author has operated upon 14 cases for epilepsy in carefully selected cases, in which the aid of the neurologist has almost always been available for diagnosis. One year, or longer, having expired since operation, the results are: Cured, 3=25 per cent. (one relapsed later); improved, 5 (in four lesion found and removed); unimproved, 4 (in two lesion found; other two none found); unknown, 2; died, 0—14. Ten of these cases were traumatic, and to this class belong the three cures and four of the cases improved by operation. The six cases which were not cured dated their injuries very far back, and a condition of chronic epilepsy had become well established.

In the non-traumatic cases the net result of operation was one improved.

The detailed reports of the author's cases are full of interest and instruction, and well deserve perusal by surgeons interested in this subject.

[1] Amer. Jour. Med. Sci., May, 1898.

### PROGRESS IN GYNÆCOLOGY AND OBSTETRICS.

**The Surgical Treatment of Pelvic Inflammation.**—Cullingworth[1] says that in suppurative pelvic cellulitis the pus should always be let out without opening the peritoneal cavity, by an incision just above Poupart's ligament, if the abscess has any tendency to point there; should the abscess be deeply seated behind the posterior layer of parietal peritoneum, an incision similar to that required for tying the external iliac artery, followed by careful dissection beneath the uplifted peritoneum, is the best. He points out that it is rare for a cellulitic abscess to point in the vagina, most of the abscesses pointing in that direction being cases of pyosalpinx or suppurating ovary. In pelvic peritonitis he thinks operation necessary in all cases attended with the formation of pus, whether in the Fallopian tube, in the ovary, or among peritoneal adhesions, and remarks that it is sometimes very difficult to say whether suppuration exists or not, the temperature being very little guide. Among cases of pelvic inflammation requiring operative treatment, in which there is no special reason to suspect suppuration, he mentions early cystic ovary (which is frequently met with as a complication of chronic salpingitis) hydrosalpinx, chronic non-suppurative salpingitis occurring in a woman belonging to the labouring class.

As regards the time for operating, he is strongly in favour of deferring operation when possible until the acute symptoms of localised peritonitis have subsided, and believes that the peritoneum after the subsidence of an acute attack of localised peritonitis possesses a certain degree of temporary immunity, and is much more tolerant of operative manipulations than at any other time, but he emphasises the point that as the protection afforded is limited in its duration, operation must not be indefinitely postponed, lest the immunising effects of the invasion should have passed off. Nicholas[2] strongly recommends vaginal section for pelvic suppuration, and says that, although a few cases cannot be cured by this method, the difficulties of abdominal section are much reduced by previous vaginal incision and drainage. M. Lucas-Championnière[3] also writes in favour of the vaginal operation in pelvic suppuration. Doyen[4] lays down the following rules as a guide to intervention in the different varieties of pelvic suppuration: First (extra-peritoneal suppuration) (*a*) phlegmon of the broad ligament—iliac incision; (*b*) abscess of inferior part of broad ligament—lateral colpotomy. Second (intra-peritoneal suppuration) (*a*) the inflammatory mass remains intrapelvic, and does not reach the brim—vaginal operation; (*b*) suppurating tumour passing the brim of the pelvis, and reaching level of umbilicus—laparotomy. By each of the above methods, vaginal and abdominal, three distinct operations may be performed: (1) Simple incision of the purulent focus; (2) ablation of the appendages leaving the uterus; (3) total castration. Macan[5] points out that when the vaginal route is employed for operation, an estimation of the difficulties to be met with is much greater than by the abdominal method, since in the former the sense of touch has to be almost exclusively relied on.

**Extra-uterine Pregnancy.**—In the Ingleby Lectures for this year, Taylor[6] maintains that abdominal or ventral pregnancy is not—as Tait and Bland Sutton have held—a mere secondary result of the broad ligament form, but that it is a variety having only very rarely any connection with a broad ligament pregnancy. As to causation, he says that normal impregnation of the ovum is not limited to the uterus, but may occur anywhere in the Fallopian tube or immediately on the exit of the ovum from the ovary; normal attachment and development is limited to the uterus only. Abnormal arrest of the impregnated ovum—whether mechanical or special—in its progress towards the uterus is the determining factor of a misplaced pregnancy. An extra-uterine pregnancy, therefore, is the consequence of the permanent arrest of a fructified ovum in its passage from the ovary to the uterus. Theoretically this may occur: (1) In the ovary; (2) in the abdominal cavity between the ovary and tube; (3) within the tube; (4) between the tube and uterus. The first and second of these are theoretical only; the fourth—in the uterine part of the Fallopian tube—may be considered as tubal, so that at the onset we have to do with one kind only, viz , tubal.

Under the heading of disturbances to its progress Taylor gives (1) early rupture of tube; (2) tubal mole; (3) later rupture of tube. Hæmorrhage from an unruptured tube containing a mole of pregnancy is the most common cause of intra-peritoneal hæmatocele. In nearly all cases of tubal pregnancy with hæmatocele the bleeding is recurrent. Taylor points out that although the hæmatocele caused by later rupture is essentially similar save in extent and severity to that of tubal mole, there are some important points of difference. The hæmorrhage of tubal mole is a more or less continuous blood-drip from the fimbriated end of the tube, varied, perhaps, by an occasional little gush of freer bleeding at irregular intervals; in later rupture of the tube the hæmorrhage is sudden and relatively copious. In tubal mole the blood has time to consolidate, and some amount of encapsulation is generally met with; in rupture of the tube, on the contrary, any encapsulation of the tube is quite exceptional. The whole condition of later rupture is more acute. Taylor next describes the three invasions of tubal pregnancy under the headings of (1) tubo-abdominal, (2) tubo-ligamentary, (3) tubo-uterine. He believes that in tubo-abdominal or ventral pregnancy, if the fœtus escapes from the tube with the amnion intact, and receives a sufficient blood supply from the maternal vessels, the pregnancy may pursue an uninterrupted course to term, and both child and placenta attain mature development within the peritoneal cavity of the mother, thus running counter to the accepted teaching of such authorities as Lawson Tait and Bland Sutton, and he brings forward cases in support of this view. He describes four different relations of the placenta to the main gestation sac in abdominal pregnancy: (1) The placenta is practically within the main gestation sac, and covered by reflexion of the amnion. (2) It has a fœtal and maternal surface of nearly equal dimensions, as in normal pregnancy, the fœtal surface being covered with the amnion and in immediate relation to the sac, while the maternal surface is growing from the spread-out remnants of the tube and peritubal structures—the back of the uterus, the broad ligament, and pelvic wall being favourite sites for such extensions of attachment. (3) The placenta remains within the tube; the tube is still recognisable, and the maternal structures are confined to the tube itself. (4) The placenta is attached to the upper wall of a broad ligament sac outside the peritoneum, and the cord passes to the child through a hole in the ligament. Advanced tubo-ligamentary pregnancy he describes under two divisions: (1) Anterior ligamentary, or sub-peritoneal abdominal pregnancy; (2) posterior ligamentary, or retro-peritoneal pregnancy. He points out that in tubo-ligamentary or broad ligament pregnancy the child is, as a rule, undermost, and the placenta above, whereas in tubo-abdominal or ventral pregnancy the child is uppermost and the placenta below.

[1] Brit. Med. Jour., Aug. 20, 1898. [2] Med. Rec., June, 1898. [3] La Semaine Medicale, July 6, 1898. [4] Lancet, Aug. 6, 1898. [5] Lancet, Aug. 6, 1898. [6] Lancet, June 4, 1898.

